# Integrating Genetic Alterations and Histopathological Features for Enhanced Risk Stratification in Non-Muscle-Invasive Bladder Cancer

**DOI:** 10.3390/diagnostics14192137

**Published:** 2024-09-26

**Authors:** Melinda Lillesand, Vebjørn Kvikstad, Einar Gudlaugsson, Ivar Skaland, Aida Slewa Johannessen, Almaz Nigatu Tesfahun, Sigmund Vegard Sperstad, Emiel A. M. Janssen, Marie Austdal

**Affiliations:** 1 Department of Pathology, Stavanger University Hospital, 4011 Stavanger, Norway; einar.gudbjorn.gudlaugsson@sus.no (E.G.); ivar.skaland@sus.no (I.S.); aida.slewa.johannessen@sus.no (A.S.J.); almaz.nigatu.tesfahun@sus.no (A.N.T.); sigmund.vegard.sperstad@sus.no (S.V.S.); emilius.adrianus.maria.janssen@sus.no (E.A.M.J.); marie.austdal@sus.no (M.A.); 2 Department of Chemistry, Bioscience and Environmental Engineering, University of Stavanger, 4021 Stavanger, Norway; 3 Department of Forensic Medicine, Oslo University Hospital, 0372 Oslo, Norway; vebkvi@ous-hf.no; 4 Department of Research, Section for Biostatistics, Stavanger University Hospital, 4011 Stavanger, Norway

**Keywords:** non-muscle-invasive bladder cancer, next-generation sequencing, genetic alterations, risk stratification

## Abstract

Background: Urothelial carcinoma presents as non-muscle-invasive bladder cancer (NMIBC) in ~75% of primary cases. Addressing the limitations of the TNM and WHO04/16 classification systems, this study investigates genetic alterations, the mitotic activity index (MAI), and immunohistochemistry (IHC) markers CK20, p53, and CD25 as better prognostic biomarkers in NMIBC. Methods: Using the Oncomine™ Focus Assay for targeted next-generation sequencing (NGS), 409 single-nucleotide variations (SNVs) and 193 copy number variations (CNVs) were identified across 287 patients with TaT1 tumors. Results: FGFR3 and PIK3CA alterations were significantly more prevalent in Ta tumors, while T1 tumors had significant ERBB2 alterations. Low-grade (LG) tumors were enriched with FGFR3 alterations, while high-grade (HG) tumors were significantly associated with ERBB2 alterations, as well as FGFR1 and CCND1 amplifications. FGFR3 alterations were linked to shorter recurrence-free survival (RFS; *p* = 0.033) but improved progression-free survival (PFS; *p* < 0.001). Conversely, ERBB2 alterations (*p* < 0.001), ERBB3 mutations (*p* = 0.044), and both MYC (*p* < 0.001) and MYCN (*p* = 0.011) amplifications were associated with shorter PFS. Survival analysis of gene sets revealed inverse associations between PIK3CA and ERBB2 (*p* = 0.003), as well as PIK3CA and MYC (*p* = 0.005), with PFS. Conclusions: In multivariate Cox regression, MAI was the strongest predictor for PFS. Integrating genetic alterations and histopathological features may improve risk stratification in NMIBC.

## 1. Introduction

Urothelial carcinoma accounts for 90% of all bladder cancer cases [1]. Approximately 75% of cases are primarily identified as NMIBC [2], whereas 50 to 70% of NMIBC patients experience tumor recurrence, and up to 30% of them progress to muscle-invasive disease (MIBC) [3]. Due to its high recurrence rate and intensive follow-up, bladder cancer is one of the most expensive cancer types to treat [4]. In NMIBC, the commonly used scoring system is the “European Association of Urology (EAU) Risk Stratification for NMIBC”. This system categorizes patients into low-, intermediate-, high-, and very-high-risk groups for treatment, considering various histopathological parameters, such as TNM stage, WHO04/16 grade, and the presence of carcinoma in situ (CIS) [5]. Bacillus Calmette–Guérin (BCG) immunotherapy is the standard treatment for intermediate- and high-risk patients with NMIBC. It prevents tumor recurrence and stage progression by activating anti-cancer immunity. However, up to 40% of patients fail to respond to BCG treatment, experiencing tumor recurrence and stage progression, while only suffering from severe side effects [3,6]. Therefore, it is important to distinguish between BCG responders and BCG non-responders. However, the current TNM and WHO04/16 classifications for bladder cancer have limitations when it comes to predicting disease course and treatment response [5]. To overcome this challenge, there is a need for an improved classification approach. Combining genetic alterations and histopathological features could improve the risk stratification of patients with NMIBC.

Urothelial carcinomas are highly heterogeneous tumors with a high mutation rate [7,8]. Previous studies in bladder cancer have found significant associations between genetic alterations, histopathological parameters, and clinical outcomes [9]. The papillary and non-papillary molecular classification system, which distinguishes tumors arising from hyperplasia and carcinoma in situ (CIS), respectively, is widely recognized for its distinct genetic alterations and clinical outcomes [10]. In general, NMIBC has a more favorable prognosis compared to MIBC, although it has a high recurrence rate [11]. It is well known that receptor tyrosine kinases (RTKs), particularly FGFRs, play a crucial role in the development of bladder cancer. Previously, it has been demonstrated that Ta LG tumors were often enriched in FGFR3 mutations and were associated with good prognosis [11,12]. However, FGFR3 mutations have also been linked to an increased risk of recurrence in NMIBC [13]. Another member of the FGFR family, FGFR1, has been less extensively studied compared to FGFR3. FGFR1 alterations have been associated with epithelial–mesenchymal transition (EMT) and a higher stage and grade in NMIBC [11,14,15]. Recently, other RTKs, such as ERBB2 and ERBB3, have gained attention in NMIBC as well. Although ERBB2 is best known for its prognostic and predictive roles in breast cancer, studies have also emphasized its prevalence in bladder cancer. In NMIBC, ERBB2 alterations were associated with a higher tumor stage and grade and linked to worse PFS [16,17,18]. Furthermore, others have identified two luminal subgroups in NMIBC: FGFR3-enriched tumors with a favorable prognosis and ERBB2/ERBB3-enriched tumors with poorer outcomes [19,20]. Moreover, cell cycle dysregulation is one of the main drivers in tumorigenesis and metastasis. The MYC family of oncogenes, including MYC and MYCN, promotes tumor growth and metastasis by upregulating key cell cycle genes, such as CCND1 [21]. Alterations in MYC and CCND1 have been frequently observed in bladder cancer, correlating with aggressive phenotypes and worse prognosis [22,23], while MYCN alterations were most common in neuroblastomas [24]. Additionally, TP53 mutations have been detected in both the luminal subtype of NMIBC and the basal subtype of MIBC, associated with adverse outcomes [25]. Previous research has shown that TP53 mutations were mutually exclusive with FGFR3 mutations in bladder cancer [26], whereas their co-occurrence with ERBB2 alterations was associated with a worse prognosis in breast cancer [27]. Genetic alterations have also impacted the phosphatidylinositol 3-kinase (PI3K) signaling pathways in NMIBC [28]. It has been reported that PIK3CA was frequently mutated in NMIBC, especially in LG tumors, and was often associated with FGFR3 mutations and better patient outcomes [29,30,31]. Our previous research has shown that high levels of CD25+ T regulatory cells (Tregs) were associated with shorter PFS in NMIBC [32]. Furthermore, studies have reported that FGFR3 and MYC overexpression led to an increase in Tregs, inducing an immunosuppressive tumor microenvironment (TME) [33], while ERBB2 overexpression was linked to a decrease in Tregs [34,35,36]. Cytokeratins are intermediate filaments that indicate the differentiation status of tumor cells. Specifically, KRT20 alterations have been associated with luminal subtypes in both NMIBC and MIBC, indicating a worse prognosis in NMIBC but a better prognosis in MIBC [10]. In our study, we used p53 and CK20 as surrogate markers for TP53 and KRT20, respectively [37].

The primary objective of this study is to investigate the association between molecular alterations, such as single-nucleotide variants (SNVs), short insertions and deletions (INDELs), and copy number variants (CNVs), and both stage progression and tumor recurrence in a retrospective, single-institution, population-based study of NMIBC patients. Additionally, we aim to enhance the risk stratification of NMIBC patients by integrating genetic alterations with histopathological, IHC, and proliferation features.

## 2. Materials and Methods

### 2.1. Cohort Description

In this population-based retrospective study, we included 349 patients diagnosed with primary papillary NMIBC at Stavanger University Hospital, Norway, between 1 January 2002 and 1 January 2011. This time period was chosen due to the availability of ethically approved data and its provision of valuable long-term follow-up, which is essential for understanding outcomes in this population. For the analysis of stage progression, all 349 patients were available, whereas in the tumor recurrence group, only 337 patients were included due to 12 cases being lost to follow-up. Additionally, 45 patient samples were excluded from the analysis due to the absence of tumor samples in the archive or insufficient material for NGS analysis. Additionally, 17 patient samples were excluded for not meeting quality requirements, leaving 287 patients for NGS data analysis in the stage progression cohort, with a median follow-up of 87 months (range: 3 to 173 months). In the tumor recurrence cohort, 277 patients were included, with a median follow-up of 71 months (range: 3 to 159 months; Table 1). The inclusion criteria for tumor recurrence and stage progression cohorts were defined in our previously published study [32]. Briefly, tumor recurrence was defined as the reappearance of a tumor three months or more after the initial diagnosis. Stage progression was defined as the diagnosis of a recurrent tumor at a higher stage, three months or more after the initial diagnosis. All patients who experienced stage progression (*n* = 21, 7%), with the exception of one, underwent radical cystectomy. The patients were censored if they underwent cystectomy. Clinical and follow-up data were retrieved from medical records at Stavanger University Hospital until 30 June 2016 [32].

### 2.2. Histopathological Features and IHC Markers

Tumor samples were processed and stained according to previously published protocols [32,38,39]. Briefly, tumors were fixed in 10% neutral buffered formalin, dehydrated, embedded in paraffin, and sectioned at 4 µm onto Superfrost Plus^®^ slides (Menzel, Braunschweig, Germany). Sections were stained with Hematoxylin, Erythrosine, and Saffron (HES) for TNM staging, WHO04/16 grading [40,41], and MAI quantification. IHC was used for the quantification of p53, CD25, CK20 [32], and androgen receptor (AR). For IHC, the following antibodies were used at the specified dilutions: p53 (DAKO, Glostrup, Denmark, clone DO-7) at 1:150, CD25 (Novocastra, Newcastle upon Tyne, UK, clone 4C9) at 1:150, CK20 (DAKO, Glostrup, Denmark, clone Ks20.8) at 1:50, and AR (Cell Marque™, Rocklin, CA, USA; clone SP107) at 1:50. Analysis of MAI, CK20, p53, and CD25 was performed according to previously published protocols [32,38,39]. AR-immunostained sections were scanned at 400× magnification using the NanoZoomer S60 (Hamamatsu, Japan) and uploaded to Visiopharm^®^ software (version 2022.09.3.12885, Hoersholm, Denmark). The area with the highest tumor percentage and least differentiation, initially selected for DNA analysis, was subsequently used for AR analysis. Automated digital image analysis (DIA) was employed to detect both positive and negative cells. Artefacts and nonspecific stains were manually removed in Visiopharm^®^. AR positivity was calculated as the percentage of AR-positive cells out of the total, with IHC positivity defined as an average of ≥50% (Figure S1 and Table S1).

### 2.3. Sample Collection and Preparation for NGS Analysis

For mutation analysis, 287 patient samples were available. Ten µm formalin-fixed, paraffin-embedded (FFPE) sections were cut from the least differentiated area with the highest tumor percentage, and DNA was extracted using the E.Z.N.A.^®^ FFPE DNA Kit (Cat. No. D3399-01, Omega Bio-tek, Inc., Norcross, GA, USA), according to the manufacturer’s protocol. Quality and quantity were measured by using NanoDrop™ 2000 Spectrophotometer (Thermo Fischer Scientific™, Waltham, MA, USA) and Qubit Fluorometric Quantification methods (Thermo Fischer Scientific™, Waltham, MA, USA). Average tumor percentage was 68% (range 5–90), and 76% of DNA samples had a tumor cell percentage (TC) over 50%. Input DNA was 10 ng. To perform NGS, the Ion Torrent Personal Genome Machine (PGM) and Ion GeneStudio S5 systems (Thermo Fischer Scientific™, Waltham, MA, USA) were used. Pooled primer libraries were prepared with the IonAmpliSeq™ Library kit 2.0 and Ion Chef System automated library preparation (Thermo Fisher™ Scientific, Waltham, MA, USA), respectively. We used the Ion Torrent™ Oncomine™ Focus Assay (Cat. No. A28548, Thermo Fischer Scientific™, Waltham, MA, USA) to detect SNVs, INDELs, and CNVs in FFPE. The Ion Torrent™ Oncomine™ Focus panel covers 35 mutational hotspots and 20 copy number genes. Furthermore, we used Ion Torrent Suite™ (version 5.18.1) and Ion Reporter™ Software (version 5.18.2.1) to analyze NGS data.

### 2.4. Variant Calling and Data Analysis

Amino acid changes identified by Ion Reporter™ Software (version 5.18.2.1)were carefully reviewed both with and without filters (Oncomine Variants, 5% CI). We cross-referenced these amino acid changes with COSMIC, My Cancer Genome^®^, ClinVar NCBI, and OncoKB™ databases. We selected variants that were confirmed as pathogenic, deleterious, or oncogenic in at least three of these databases. The amino acid change variants needed to meet a coverage depth limit of detection (LOD) of ≥500× and a variant allele frequency (VAF) of ≥5%. The average VAF was 38%, and the average coverage depth was 1678. Among all genetic aberrations (*n* = 287), we identified 411 annotated amino acid changes comprising 409 SNVs and 2 INDELs alterations (Table S2). Furthermore, we investigated CNVs using a threshold of 15 copies for TC ≤ 10%, 10 copies for 10% < TC ≤ 20%, and 7 copies for TC > 20%. Among 101 patients, we identified 193 CNVs (Table S3). Out of the 52-gene panel from the Ion Torrent™ Oncomine™ Focus panel, a total of 29 genes with SNVs and CNVs were identified (Figure S2).

### 2.5. Statistics

All statistics were performed in R (version 4.3.2). SNVs and CNVs were analyzed together to gain a more comprehensive understanding of the genetic alteration landscape, collectively referred to as alterations or genetic alterations when both were present in a sample. We used the maftools open-source R package for comprehensive analysis of genetic alterations within the clinical context [42], and generated an oncoplot in the style of cBioPortal [43,44,45]. Missing values were excluded from mutational signature analysis, apolipoprotein B mRNA editing enzyme, catalytic polypeptide-like cytidine deaminases (APOBEC) signature analysis, clinical enrichment analysis, and interaction analysis. Statistical significance was determined by a *p*-value less than 0.01 and a false discovery rate (FDR)-adjusted *p*-value less than 0.05. By default, genes with alterations were considered only if they were present in a minimum of five samples. The mutational signature and APOBEC signature analyses were based on the 96 single-base substitution (SBS) classification. The trinucleotideMatrix function was used to extract adjacent 5′ and 3′ bases neighboring the mutated site, resulting in a frequency matrix with dimensions of 238 × 96. In the mutational signature analysis, the estimateSignatures function was used to determine the optimal number of signatures based on the cophenetic correlation coefficient. The extractSignatures function was used to extract mutational signatures from the trinucleotide context using non-negative matrix fractionization. Thereafter, the extracted mutational signatures were compared to the 30 experimentally validated COSMIC signatures using cosine similarity values. Furthermore, signatureEnrichment analysis followed by Fisher’s exact test was conducted to assess the association between genes and signatures [7,43]. In the APOBEC signature analysis, tCw-to-tTw or to-tGw alterations were considered as APOBEC mutation motifs within a ±20 nucleotide range. APOBEC enrichment scores were calculated and analyzed by the one-way Fisher’s test designed for APOBEC mutation analysis [43,46]. In the clinical enrichment analysis, the associations between genetic alterations and clinical features, MAI and IHC markers, were investigated. Clinical variables, including tumor recurrence, stage progression, TNM stage, and WHO04/16 grade, were categorical variables. Proliferation marker MAI and IHC markers CK20, p53, and CD25 were dichotomized based on previously published thresholds [32,39]. Contingency tables were generated for every categorical variable, followed by Fisher’s exact test [43]. In the interaction analysis of genetic alterations, the exclusivity or co-occurrence between gene sets was analyzed using Fisher’s exact test [43]. Furthermore, the maftools::mafSurvival function was employed for log-rank survival analysis to investigate the association between genetic alterations and PFS and RFS. Additionally, we utilized maftools::survGroup to identify gene sets linked to survival outcomes. Patients with undetected genetic alterations were included, treating them as missing values to ensure unbiased results. Mutated genes were only considered if they were detected in at least five samples. Cox multivariate regression analysis was conducted on variables significant in univariate analysis (*p*-values less than 0.05), with significance determined at *p*-values less than 0.05.

## 3. Results

We identified FGFR3 (45%) and PIK3CA (24%) as the most frequent gene variants, while AKT1 (20%), ERBB2 (12%), MYCN (12%), and CCND1 (11%) were the most amplified genes. In FGFR3, the most common amino acid changes were p.Ser249Cys (54%), p.Arg248Cys (14%), and p.Tyr373Cys (20%). In PIK3CA, prevalent amino acid changes were p.Glu545Lys (31%), p.Glu542Lys (19%), and p.His1047Arg (13%).

### 3.1. Histopathological Parameters and IHC Markers

In our molecular analysis, 287 patients were included in the stage progression (*n* = 21, 7%) cohort, and 277 patients were included in the tumor recurrence (*n* = 145, 52%) cohort (Table 2).

### 3.2. Association between Genetic Alterations and TNM Stage and WHO04/16 Grade

In our molecular analysis, we included 287 patients, with no mutations detected in 17 of them, leaving a cohort of 270 patients for further analysis. Among these, 169 had SNVs, 32 had CNVs, and 69 had a combination of both. In the 270 patients, 80% had Ta tumors, 20% had T1 tumors, 63% had LG tumors, and 37% had HG tumors. In clinical enrichment analysis, we observed distinct genetic associations among Ta, T1, LG, and HG tumors. Ta tumors were significantly associated with FGFR3 and PIK3CA alterations, while T1 tumors were significantly associated with ERBB2 alterations (FDR < 0.05). On the other hand, LG tumors were significantly enriched with FGFR3 alterations, while HG tumors were significantly associated with ERBB2 alterations and FGFR1 and CCND1 amplifications (FDR < 0.05; Table 3).

### 3.3. Association between Genetic Alterations and MAI and IHC Markers

**Figure 1:**
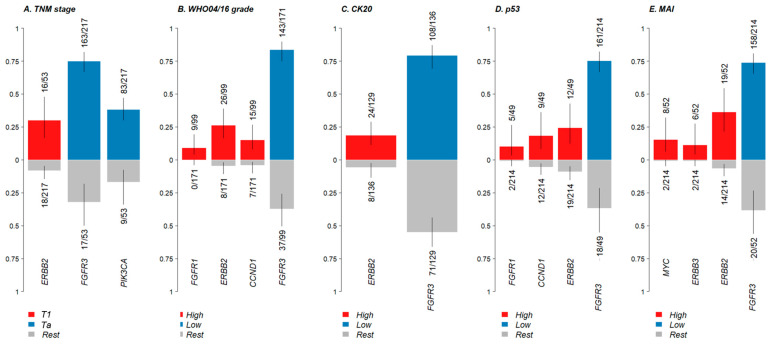
Bar plots show the relationship between genetic alterations and (**A**) TNM stage, (**B**) WHO04/16 grade, (**C**) CK20, (**D**) p53, and (**E**) MAI (FDR < 0.05). Each bar represents the ratio of mutated samples to total samples, with error bars indicating a 95% confidence interval (CI). The y-axis denotes the fraction of samples categorized by TNM stage, WHO04/16 grade, CK20 positivity, p53, and MAI.

In the clinical enrichment analysis, significant associations were observed between genetic alterations and MAI (*n* = 266), as well as IHC markers CK20 (*n* = 265), p53 (*n* = 263), and CD25 (*n* = 264). CK20 low (negative) was linked to FGFR3 alterations, whereas CK20 high (positive) was associated with ERBB2 alterations (FDR < 0.05). Additionally, low (<15%) p53 expression was associated with FGFR3 alterations, while high (≥15%) p53 expression was associated with ERBB2 alterations and FGFR1 and CCND1 amplifications (FDR < 0.05). Regarding proliferation, low (≤15) MAI was linked to FGFR3 alterations, while high (>15) MAI was associated with MYC amplifications and alterations in ERBB2 and ERBB3 mutations (FDR < 0.05). No significant differences in gene alterations were observed between low and high CD25 groups (Table 4 and Figure 1).

### 3.4. Interaction Analysis of Genetic Alterations

**Figure 2:**
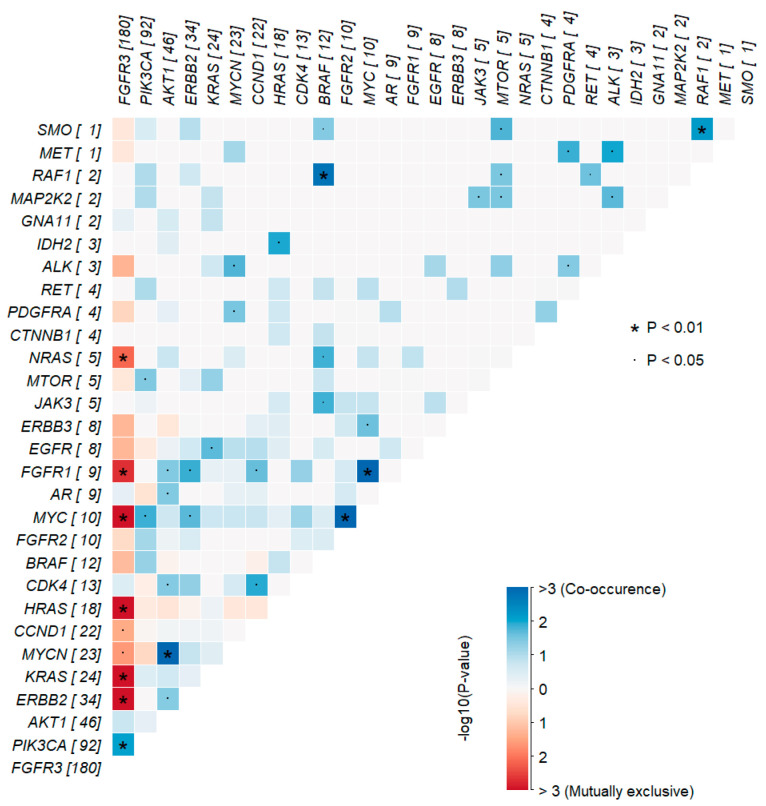
Mutually exclusive and co-occurring genetic alterations in NMIBC. The blue color indicates a tendency toward co-occurrences and red indicates mutual exclusivity. Visualization created with maftools.

In the interaction analysis of genetic alterations, we found that FGFR3 alterations were mutually exclusive with amplifications of FGFR1, MYC, MYCN, and CCND1, as well as alterations in ERBB2 and KRAS, and mutations in HRAS and NRAS (*p* < 0.05; Figure 2).

### 3.5. Genetic Alteration Landscape

**Figure 3:**
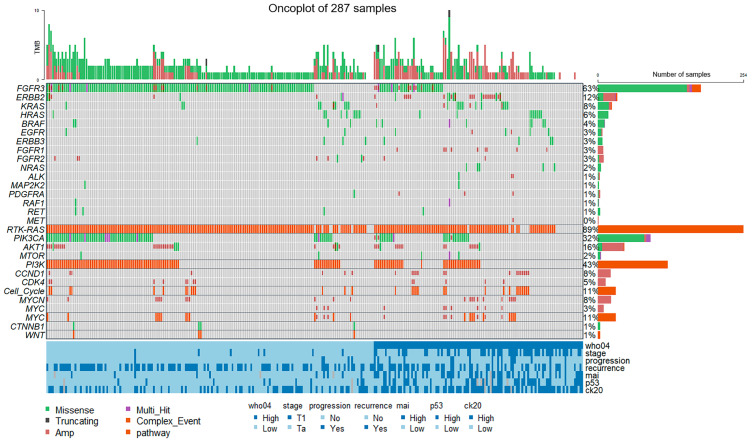
The oncoplot displays the genetic alterations landscape of the NMIBC cohort. Genes are ordered by mutation frequency, while samples are arranged according to WHO04/16 grade, as indicated by the annotation bar at the bottom. The sidebar plot illustrates the percentage distribution of altered genes within the cohort. The top bar plot indicates the tumor mutational burden, representing the number of mutations and CNVs per sample. Multi-hit events, marked in purple, indicate multiple SNVs within the same patient, while complex events, marked in red, indicate the presence of both SNVs and CNVs in the same patient. Oncogenic signaling pathways, including RTK-RAS, PI3K, cell cycle, MYC, and WNT, are also presented in the oncoplot, with the respective genes and their percentage representation in each pathway.

In the oncoplot analysis, 287 patients were included. The oncoplot visually represents SNVs (marked in green) and CNVs (marked in maroon) across samples in a matrix format. Each row in the matrix represents a gene, and each column represents a sample. The sidebar plot displays the percentage distribution of genetic alterations within our patient group. FGFR3 alterations were most prevalent, affecting 63% of the patients in our study. In Ta LG tumors (*n* = 168), we observed a total of 275 SNVs and 78 CNVs, whereas in T1 HG tumors (*n* = 53), there were 50 SNVs and 53 CNVs. Compared to T1 HG tumors, Ta LG tumors exhibited higher frequencies of alterations in specific genes, including FGFR3 (82%), PIK3CA (39%), and AKT1 (17%). Conversely, T1 HG tumors showed higher frequencies of alterations in most other genes. Additionally, alterations in ALK and MET, amplifications of FGFR1, and mutations in NRAS and SMO were exclusively observed in T1 HG tumors (Figure 3).

### 3.6. Analysis of Stage Progression and Tumor Recurrence

**Figure 4:**
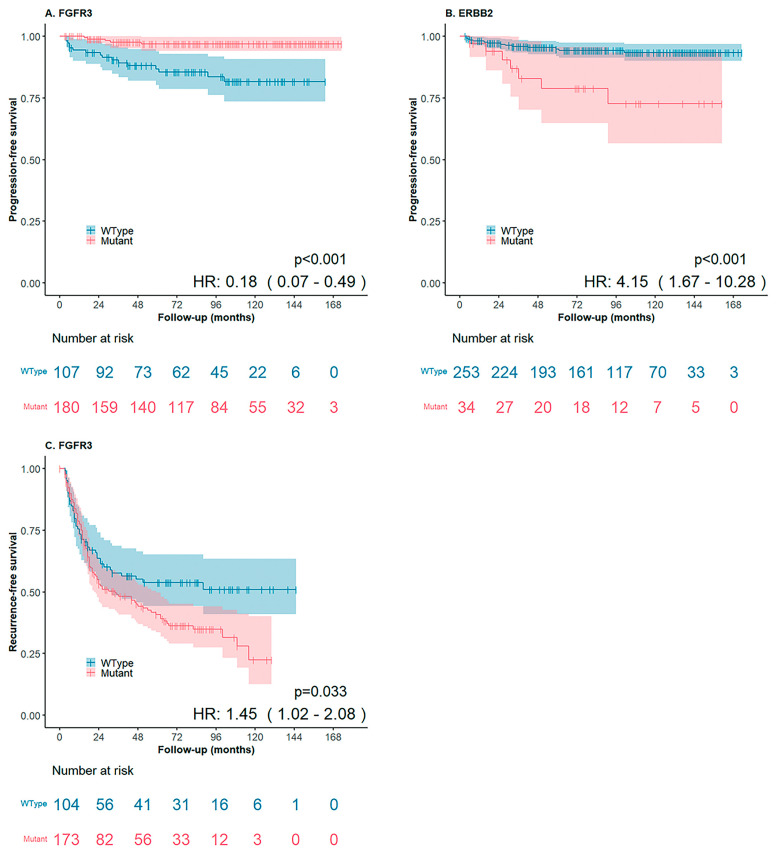
(**A**) FGFR3 was significantly associated with better PFS (*p* < 0.001). (**B**) ERBB2 was significantly associated with shorter RFS (*p* < 0.001). (**C**) FGFR3 was significantly associated with shorter RFS (*p* < 0.033).

In the analysis of stage progression, 287 patients were included, with 7% experiencing progression. In the log-rank survival analysis, FGFR3 alterations were significantly associated with better PFS (HR: 0.2, 95% CI: 0.1–0.5, *p* < 0.001). Conversely, genetic alterations in ERBB2 (HR: 4.2, 95% CI: 1.7–10.3, *p* < 0.001), ERBB3 mutations (HR: 4.0, 95% CI: 0.9–17.1, *p* = 0.044), and MYC and MYCN amplifications (HR: 6.5, 95% CI: 1.9–22.2, *p* < 0.001, and HR: 3.4, 95% CI: 1.3–9.4, *p* = 0.011, respectively) were significantly associated with shorter PFS (Figure 4). Survival analysis of gene sets revealed associations between PIK3CA and ERBB2 (HR: 5.2, 95% CI: 1.5–17.7, *p* = 0.003) and PIK3CA and MYC (HR: 6.3, 95% CI: 1.5–27.1, *p* = 0.005), both linked to shorter PFS. Furthermore, in multivariate Cox regression analysis, MAI emerged as the strongest predictor of progression (HR: 17.5, 95% CI: 5.7–53.1, *p* < 0.001). In the analysis of tumor recurrence, 277 patients were included, with 52% experiencing recurrence. In log-rank survival analysis, patients with FGFR3 alterations had significantly shorter RFS (HR: 1.5, 95% CI: 1.02–2.1, *p* = 0.033; Figure 4). No multivariate analysis was conducted for RFS, as FGFR3 was the only gene significantly associated with RFS.

### 3.7. Pathway Analysis

In our cohort (*n* = 287), most patients had genetic alterations in the RTK-RAS pathway (*n* = 254, 89%) and PI3K pathway (*n* = 122, 43%). The MYC and cell cycle pathways were each affected in 11% of patients (*n* = 31), while the WNT pathway was only impacted in 1% of patients (*n* = 4). In the RTK-RAS pathway, 16 genes were affected in our cohort (Figure 3). In the log-rank survival analysis, there was no significant association between the RTK-RAS pathway and either RFS or PFS. Alterations in RTKs, including FGFRs, ERBBs, PDGFRA, RET, ALK, and MET, were identified in 78% of patients (*n* = 225). In the log-rank survival analysis, RTKs showed a significant association with worse RFS (*p* = 0.013). HRAS, KRAS, NRAS, BRAF, and RAF1 are key components of the mitogen-activated protein kinase (MAPK) pathway, and alterations in these genes were identified in 18% of patients (*n* = 52). No significant associations were observed between the key components of the MAPK pathway and either RFS or PFS in our study. In the PI3K pathway, PIK3CA, AKT1, and MTOR genes were affected in our cohort. In our study, we did not find any significant association between the PI3K pathway and either RFS or PFS. The MYC pathway, including MYC and MYCN transcription factors, showed a significant association with PFS (*p* < 0.001). Conversely, the cell cycle pathway, which includes CCND1 and CDK4, was not associated with either RFS or PFS.

### 3.8. Mutational Signature and APOBEC Enrichment Analysis

Mutational signatures are distinct patterns of six single-base substitutions (SBSs) and their neighboring nucleotides, linked to specific mutational processes. Using eight as the best-fit rank based on the decreasing cophenetic correlation coefficient, we identified eight mutational signatures associated with COSMIC signatures, including SBS1, 2, 3, 4, 7, 15, 16, and 25. Among these, SBS2 had the highest cosine similarity score (0.856), associated with the APOBEC mutational signature. In the signature enrichment analysis, SBS2 was significantly associated with PIK3CA alterations (*n* = 29, FDR < 0.05; Table 5 and Figure S3). Additionally, we confirmed these results using APOBEC enrichment analysis, where APOBEC-related alterations were enriched in 14% of patients (25/175) with an APOBEC enrichment score greater than 2. Among these APOBEC-enriched samples, 21 (84%) had PIK3CA alterations (*p* < 0.001). No significant association was found between APOBEC-related alterations and either RFS or PFS.

### 3.9. Separate Analysis of Ta and T1 Tumors

In our study cohort, 80% of patients had Ta tumors, while 20% had T1 tumors. Among the Ta tumors, 74% were LG, and 26% were HG. Conversely, in T1 tumors, 12% were LG, and 88% were HG. In Ta tumors, the most frequent gene variants were FGFR3 (48%) and PIK3CA (26%), and the most common amplifications were AKT1 (24%), FGFR3 (13%), MYCN (11%), and CCND1 (11%). In T1 tumors, the most frequent gene variants were FGFR3 (23%), PIK3CA (13%), and ERBB2 (13%), and the most amplified genes were ERBB2 (23%), MYCN (13%), and CCND1 (11%). Ta HG tumors were enriched with ERBB2 alterations and FGFR1 and CCND1 amplifications, whereas FGFR3 alterations were prevalent in both Ta LG and T1 LG tumors (Table 6). Regarding clinical outcomes, 4% (*n* = 8) of patients with Ta tumors experienced stage progression, and FGFR3 alterations were associated with better PFS (*p* = 0.003). Conversely, alterations in PIK3CA (*p* = 0.025) and ERBB2 (*p* = 0.045), as well as ERBB3 mutations (*p* = 0.025) and amplifications of MYC (*p* < 0.001), FGFR1 (*p* = 0.044), and CCND1 (*p* = 0.034), were associated with worse PFS in Ta tumors. For T1 tumors, 22% (*n* = 13) of patients showed stage progression, and MYCN was significantly associated with worse PFS (*p* < 0.001). For tumor recurrence, Ta tumors had a 52% recurrence rate, compared to 45% for T1 tumors. In T1 tumors, alterations in AKT1 (*p* = 0.010) and FGFR3 (*p* = 0.032) were associated with worse RFS, but no significant association was observed between RFS and genetic alterations in Ta tumors.

### 3.10. Association between BCG Immunotherapy and Genetic Alterations

Out of 287 patients, 79 received BCG immunotherapy and were included in our molecular analysis to evaluate treatment response. The overall intravesical BCG treatment rate was low, with only 28% (*n* = 79) treated, reflecting a 4:1 ratio of Ta to T1 stages in our study. BCG treatment included both induction and maintenance phases. Among the 79 treated patients, 92% completed the induction course, defined as receiving at least five installations. The median number of BCG installations was 15 (range: 1 to 24). Of the 79 patients, 44% had T1 tumors, and 70% had HG tumors. Tumor recurrence occurred in 66% of patients, with 13% experiencing progression to a higher stage. BCG non-responders were defined as patients who experienced tumor recurrence and/or stage progression, while BCG responders were those who did not. Interestingly, we identified eight AR amplifications and one H875Y mutation in the BCG-treated cohort. To test these findings, AR IHC was performed on seven available samples with AR mutations, as well as on parallel controls without AR mutations. Of the seven samples with AR alterations, six were IHC-positive (≥50%). In contrast, all samples without AR alterations were IHC-negative (<50%). In log-rank survival analysis, genetic alterations in FGFR3, AKT1, and AR were significantly associated with shorter RFS (*p* = 0.010, 0.004, and 0.007, respectively), while PIK3CA alterations were linked to shorter PFS (*p* = 0.044) in the BCG-treated cohort. When analyzing mutational signatures in these patients, no statistically significant, differentially mutated genes were identified. This result is likely due to the small sample size and limited number of mutations.

## 4. Discussion

We aimed to investigate the association between genetic alterations and histopathological and IHC features to improve the risk stratification of patients with NMIBC.

In our study, distinct genetic profiles within Ta, T1, LG, and HG tumors were identified. We found that FGFR3 alterations were significantly associated with Ta LG tumors. This aligns with prior studies, showing that FGFR3 alterations were more prevalent in less aggressive, early-stage bladder cancers [11,47]. Additionally, our results showed a significant association between ERBB2 alterations and T1 HG tumors. Clinical enrichment analysis further revealed that ERBB2 alterations were associated with high expression of CK20, p53, and a high proliferation rate. Hedegaard et al. similarly reported that Ta LG tumors were enriched with FGFR3 alterations, while T1 HG tumors were enriched with KRT20, TP53, and ERBB2 alterations [19]. Although PIK3CA alterations were more common in Ta tumors in our cohort, as confirmed by others [28], we found no significant association with the WHO04/16 grade. Similarly, Duenas et al. also did not find any association between PIK3CA alterations and tumor grade. However, they found a higher frequency of PIK3CA alterations in T1 tumors, with a Ta:T1 ratio of 1:1, suggesting an important role in tumor aggressiveness [29]. Discrepancies between our findings and theirs may be due to the differences in the ratio of Ta:T1 stages, which was 4:1 in our study. Moreover, FGFR1 and CCND1 alterations were found in 3% and 8% of our cohort, respectively. FGFR1 alterations promote tumor invasion through EMT mechanisms [48], while CCND1 regulates cell cycle progression through cyclin pathways [49]. In our cohort, HG tumors had a significantly higher frequency of FGFR1 and CCND1 alterations compared to LG tumors. Previous studies have also confirmed that FGFR1 and CCND1 alterations were linked to HG tumors [15,50]. In somatic interaction analysis, FGFR1 and CCND1 were found to be significantly mutually exclusive with FGFR3 alterations. These findings may indicate that alterations in FGFR1 and CCND1 represent early events in tumorigenesis and act as drivers for tumor progression in NMIBC.

In the assessment of stage progression, FGFR3 alterations were associated with improved PFS, while alterations in ERBB2, MYC, and MYCN were linked to shorter PFS. Others have also reported that FGFR3 expression is associated with better PFS in NMIBC, while frequent amplifications of ERBB2 and MYC were linked to poor prognosis [51,52]. In line with other publications, our results revealed mutual exclusivity between ERBB2 and FGFR3 [17]. To the best of our knowledge, limited literature has investigated the association between MYCN and patient outcomes in NMIBC. MYCN, a transcription factor in the MYC family, is known to be frequently amplified in neuroblastoma, causing an aggressive disease course [53]. In NMIBC, Hedegaard et al. published that MYCN alterations were associated with cell cycle process and a more aggressive luminal subtype [19]. Consistent with other findings, we could not find a significant association between PIK3CA alterations alone and PFS [30]. However, PIK3CA in combination with ERBB2 or MYC was inversely associated with PFS. Although PIK3CA alterations are common in bladder cancer, their prognostic significance is unclear and may depend on interactions with other genetic alterations [54]. These findings suggest that identifying gene sets may more accurately capture the biological complexity of patient outcomes, providing greater predictive power than single-gene analyses. In the assessment of tumor recurrence, FGFR3 alterations were significantly associated with shorter RFS. Some studies have also confirmed that FGFR3 alterations were linked to worse RFS, but better PFS [52,55]. Additionally, RTKs within the RTK-RAS pathway, including FGFRs, ERBBs, PDGFRA, RET, ALK, and MET, were also linked to shorter RFS. This finding may be due to a potential overrepresentation of FGFRs among RTKs. In summary, FGFR3 alterations play a crucial role in tumor initiation but may not be the primary drivers of stage progression. Instead, there seems to be a potential shift toward ERBB2 dominance in higher-stage tumors.

Recent efforts have focused on the molecular classification of NMIBC. Hurst et al. emphasized the need to distinguish between Ta (non-invasive) and T1 (submucosal invasive) tumors due to significant molecular heterogeneity within these stages [9]. Our analysis identified two subgroups in Ta tumors: one with FGFR3 alterations linked to LG tumors and favorable outcomes, and another with alterations in ERBB2, PIK3CA, and ERBB3 mutations, and FGFR1, CCND1, and MYC amplifications, associated mostly with HG tumors and poorer prognosis. In T1 tumors, FGFR3 alterations were linked to worse RFS, and MYCN amplifications were associated with worse PFS. Our findings are similar to the molecular subtypes identified by the Aarhus group classification and the Lund taxonomy applied to NMIBC: Class I/UroA is enriched with FGFR3, Class 2a and 2b with ERBB2, ERBB3, and FGFR1, and UroC/Genomically Unstable (GU) with MYCN and PIK3CA alterations [56].

The EAU guidelines recommend intravesical BCG as the primary treatment for intermediate- and high-risk NMIBC [5]. However, the biological mechanisms driving tumor recurrences are still not well understood. In our BCG-treated patient group, FGFR3 and PIK3CA alterations were significantly associated with shorter RFS and PFS, respectively. Previous publications have demonstrated that downregulation of FGFR3 was associated with BCG responders [57], while FGFR3 overexpression was linked to BCG non-responders [58]. In accordance with prior studies, our results suggest a significant association between FGFR3 and PIK3CA alterations, and poor response to BCG treatment [59,60]. Interestingly, our study found that genetic alterations in AR were associated with shorter RFS, a surprising result given that AR amplifications are typically associated with castration-resistant prostate cancer [61]. However, research has demonstrated that AR and the androgen signaling pathway play a role in the etiology and progression of bladder cancer [62], and combining antiandrogens with BCG immunotherapy could improve its efficacy [63,64,65]. Furthermore, the current BCG shortage and issues of BCG unresponsiveness emphasize the urgent need for better treatment options. Frequent alterations in RTKs make them promising targets for more personalized treatments [66]. Upon analyzing amino acid variants of FGFR3, the most common variants were Ser249Cys and Arg248Cys in the extracellular domain, and Tyr373Cys in the transmembrane domain, causing constant downstream signaling without the presence of ligands. Recently FDA-approved, erdafitinib, a pan-FGFR (FGFR1-4) tyrosine kinase inhibitor (TKI), has shown efficacy in treating advanced bladder cancer and intermediate- and high-risk NMIBC by blocking tyrosine kinase signaling [67,68]. In our cohort, ERBB2 amplification was more prevalent compared to missense alterations, consistent with observations in other cancer types [69,70]. Despite success in breast and gastric cancers, clinical trials with anti-ERBB2 treatments have shown limited efficacy in bladder cancer. Furthermore, it has been previously reported that inhibition of FGFR3 can trigger compensatory ERBB2 signaling, emphasizing the importance of targeting both pathways simultaneously [71]. In our study, the most frequent variants of PIK3CA were Glu545Lys, Glu542Lys, and His1047Arg. These alterations are associated with the catalytic subunit encoded by the PIK3CA gene and can lead to constant activation of the PI3K pathway [72]. In general, PI3K inhibitors, such as alpelisib, are effective treatment options in bladder cancer but can cause very severe side effects [73].

In the cancer genome, SBS signatures reveal distinct patterns of alterations associated with specific mutagenic processes. In our analysis of mutational signatures, we observed that C > T (35%) was the most frequent base substitution linked to the APOBEC mutational signature. It has been similarly documented that in NMIBC, the APOBEC mutational signature contributes up to 30% of all alterations [74,75]. APOBEC enzymes induce hypermutation by deaminating cytosine bases, resulting in C > T and C > G substitutions [76]. Moreover, we found that the APOBEC mutational signature was significantly associated with PIK3CA alterations. In bladder cancer, previous reports have also observed that C > T base substitutions were most common in FGFR3 and PIK3CA [77]. Additionally, we did not find a significant association between the APOBEC mutational signature and either RFS or PFS. However, others have demonstrated that enrichment of APOBEC mutational signatures was linked to high-risk NMIBC and poor prognosis [19,20]. Furthermore, we also identified mutational signatures, such as SBS15 and SBS7, which were associated with defective DNA mismatch repair and smoking mutational processes, respectively. However, our mutational signature results must be interpreted carefully, as SBS signatures are based on whole-genome sequencing (WGS) data, whereas our study used a targeted NGS panel, resulting in fewer alterations.

To the best of our knowledge, our study is among the largest single-institution, population-based studies focused exclusively on patients with NMIBC and is the largest to separately analyze Ta tumors (*n* = 227). Although we examined a limited number of genes, we validated previous findings and identified key tumor-driver genes in NMIBC. A significant strength of our research is the utilization of a well-established NGS panel that is routinely used in diagnostic settings. This enhances the clinical relevance of our findings and underscores their potential for integration into standard clinical practice.

In NMIBC, differentiating between LG and HG tumors is challenging because there are no standardized and reproducible methods available. In our research, we could differentiate between Ta and T1, as well as LG and HG tumors, based on their distinct molecular profiles. Previously, both our study and others have shown a significant association between IHC markers and proliferation features, and TNM stage, WHO04/16 grade, and patient outcomes [32,39,78]. We further established that IHC markers and MAI were significantly linked to distinct genetic alterations. Confirming our earlier findings, MAI was the best predictor for stage progression and was associated with a more aggressive molecular phenotype. Moreover, we also found that distinct molecular profiles were associated with RFS and PFS. In summary, our results confirm that integrating histopathological, IHC, and proliferation features with genetic alterations enhances the objective and reproducible classification of patients into appropriate risk groups. To enhance the robustness of our findings, validation with larger gene panels, including genes involved in cell cycle regulation (CDKN2A and RB1), chromatin remodeling (KDM6A, ARID1A, and STAG2), DNA repair (ERCC2), tumor suppression (MTAP), and metabolic pathways (PPARG), as well as expanded cohorts, is warranted.

## 5. Conclusions

Integrating genetic alterations, histopathological features, and IHC markers enhances risk stratification in NMIBC. Our single-institution study with extended follow-up identified distinct genetic profiles for Ta, T1, LG, and HG tumors. FGFR3 alterations were prevalent in Ta LG tumors, whereas ERBB2, FGFR1, and CCND1 alterations were significantly associated with Ta HG tumors. FGFR3 alterations were linked to better PFS in Ta tumors but worse RFS in T1 tumors. Additionally, ERBB2 alterations, ERBB3 mutations, and MYC and MYCN amplifications were linked to poorer outcomes. In BCG-treated patients, tumors enriched with FGFR3 and PIK3CA alterations were more likely to show treatment failure. These findings could be integrated into routine diagnostics, but further validation with comprehensive gene panels and larger cohorts is needed.

## Figures and Tables

**Table 1:** Summary of available patient data for NGS analysis.

NGS Data Analysis	Number of Patients
Patients diagnosed with TaT1 tumor	349
Patient samples that were missing:	45
- Absence of patient samples in the archive	19
- Insufficient material for NGS analysis	26
Patient samples did not fulfill QC requirements	17
Patient samples included for NGS data analysis:	287
- No mutations	17
- SNVs	169
- CNVs	32
- Both SNVs and CNVs	69
Patient samples for stage progression analysis	287
Patient samples included for tumor recurrence analysis	277

**Table 2:** Summary of histopathological features and investigated IHC markers in association with stage progression and tumor recurrence, along with the corresponding threshold values.

Histopathological Parameters		Stage Progression		HR	Tumor Recurrence		HR
		N Events (%)	*p*-Value	95% CI	N Events (%)	*p*-Value	95% CI
Age	<72	5/144 (3)	0.004	4.0 (1.5–10.9)	68/139 (49)	0.005	1.6 (1.2–2.2)
	>72	16/143 (11)			77/138 (56)		
Sex	Male	18/209 (9)	0.165	0.4 (0.1–1.5)	103/201 (51)	0.766	1.0 (0.7–1.4)
	Female	3/78 (4)			42/76 (55)		
Stage	Ta	8/227 (3)	<0.001	7.1 (3.0–17.1)	118/222 (53)	0.917	0.9 (0.7–1.6)
	T1	13/60 (22)			27/55 (49)		
WHO04	Low	5/175 (3)	<0.001	5.6 (2.1–15.4)	92/169 (54)	0.865	1.0 (0.7–1.5)
	High	16/112 (14)			53/108 (49)		
Multifocality	No	7/170 (4)	0.013	3.1 (1.2–8.0)	72/164 (44)	<0.001	1.8 (1.3–2.5)
	Yes	11/90 (12)			56/87 (64)		
CIS	No	16/264 (6)	0.002	4.2 (1.6–11.6)	135/255 (53)	0.801	0.9 (0.5–1.8)
	Yes	5/23 (12)			10/22 (45)		
BCG	No	11/208 (5)	0.033	2.5 (1.04–5.8)	93/198 (47)	0.002	1.7 (1.2–2.4)
	Yes	10/79 (13)			52/79 (66)		
MAI	≤15	6/225 (3)	<0.001	11.6 (4.5–29.8)	110/218 (50)	0.107	1.4 (0.9–2.0)
	>15	15/58 (26)			34/55 (62)		
CK20	Negative	6/144 (4)	0.081	2.3 (0.9–6.1)	71/136 (52)	0.388	1.2 (0.8–1.6)
	Positive	13/138 (9)			71/136 (52)		
CD25	<1.3	4/141 (3)	0.004	4.3 (1.5–12.9)	73/137 (53)	0.949	1.0 (0.7–1.4)
	≥1.3	17/140 (12)			71/134 (53)		
p53	<15	10/226 (4)	<0.001	4.6 (1.9–11.0)	116/218 (53)	0.789	0.9 (0.6–1.5)
	≥15	10/54 (18)			26/52 (50)		

HR: hazard ratio; CI: confidence interval.

**Table 3:** Results of the clinical enrichment analysis in the association between genetic alterations and TNM stage and WHO04/16 grade.

TNM Stage	Genes	Comp.	n Altered	Ref.	n Altered	*p*-Value	OR	OR Low	OR High	FDR
WHO Grade		Group	Comp. Group (%)	Group	Ref. Group (%)					
Stage	FGFR3	Ta	163/217 (75)	T1	17/53 (32)	<0.001	6.34	3.18	13.08	<0.001
	PIK3CA	Ta	83/217 (38)	T1	9/53 (17)	0.003	3.02	1.36	7.40	0.018
	ERBB2	T1	16/53 (30)	Ta	18/217 (8)	<0.001	4.74	2.06	10.88	<0.001
Grade	FGFR3	Low	143/171 (84)	High	37/99 (37)	<0.001	8.47	4.64	15.85	<0.001
	ERBB2	High	26/99 (26)	Low	8/171 (5)	<0.001	7.20	2.99	19.31	<0.001
	FGFR1	High	9/99 (9)	Low	0/171 (0)	<0.001	Inf	3.63	Inf	<0.001
	CCND1	High	15/99 (15)	Low	7/171 (4)	0.002	4.16	1.53	12.55	0.009

OR: odds ratio; FDR: false discovery rate; Ref. group: reference group; Comp. group: comparison group. The reference group was the baseline for comparison, while the comparison group was evaluated against it to identify differences.

**Table 4:** Results of the clinical enrichment analysis in the association between genetic alterations and MAI, as well as IHC markers CK20 and p53.

IHC	Gene	Comp.	n Altered	Ref.	n Altered	*p*-Value	OR	OR Low	OR High	FDR
		Group	Comp. Group (%)	Group	Ref. Group (%)					
CK20	FGFR3	Low	108/136 (79)	High	71/129 (55)	<0.001	3.14	1.78	5.64	<0.001
	ERBB2	High	24/129 (19)	Low	8/136 (6)	0.002	3.64	1.50	9.78	0.017
p53	FGFR3	Low	161/214 (75)	High	18/49 (37)	<0.001	5.19	2.58	10.74	<0.001
	FGFR1	High	5/49 (10)	Low	2/214 (14)	0.003	11.87	1.87	128.61	0.023
	ERBB2	High	12/49 (24)	Low	19/214 (9)	0.005	3.31	1.34	7.91	0.026
	CCND1	High	9/49 (18)	Low	12/214 (6)	0.007	3.76	1.31	10.49	0.026
MAI	FGFR3	Low	158/214 (74)	High	20/52 (38)	<0.001	4.48	2.28	9.01	<0.001
	ERBB2	High	19/52 (37)	Low	14/214 (7)	<0.001	8.13	3.49	19.42	<0.001
	MYC	High	8/52 (15)	Low	2/214 (1)	<0.001	18.95	3.62	188.86	<0.001
	ERBB3	High	6/52 (12)	Low	2/214 (1)	0.001	13.63	2.34	142.47	0.004

OR: odds ratio; FDR: false discovery rate; Ref. group: reference group; Comp. group: comparison group. The reference group was the baseline for comparison, while the comparison group was evaluated against it to identify differences.

**Table 5:** Summary of mutational signatures associated with SBS signatures, mutational processes, and genes.

Sig.	*n*	SBS Sig.	Mutational Process	Cosine Sim.	Gene	*p*-Value	OR	OR Low	OR High	FDR
1	28	15	Defective DNA MMR	0.710	FGFR3	0.002	10.99	1.73	459.96	0.017
2	29	2	APOBEC (C > T)	0.856	PIK3CA	<0.001	65.40	10.34	2692.31	<0.001
3	15	1	Spontaneous deamination	0.614	EGFR	<0.001	38.14	4.89	460.43	0.002
			of 5-metylcytosine		KRAS	0.008	5.62	1.36	20.60	0.047
4	30	4	Smoking	0.483	HRAS	<0.001	7.10	2.19	22.47	0.004
5	63	3	Defective DNA-DSB repair	0.231	FGFR3	<0.001	Inf	8.64	Inf	<0.001
6	19	7	UV exposure	0.801	ERBB2	<0.001	9.07	2.58	30.57	0.003
7	31	16	Unknown	0.565	No association					
8	23	25	Unknown	0.393	PIK3CA	0.006	3.48	1.31	9.94	0.037

Sig.: signatures; Cosine sim.: cosine similarity; OR: odds ratio; FDR: false discovery rate.

**Table 6:** Results of the clinical enrichment analysis in the association between genetic alterations and WHO04/16 grade when analyzing Ta and T1 tumors separately.

Stage	Genes	Comp.	n Altered	Ref.	n Altered	*p*-Value	OR	OR Low	OR High	FDR
		Group	Comp. Group (%)	Group	Ref. Group (%)					
Ta	FGFR3	LG	137/164 (84)	HG	26/53 (49)	<0.001	5.22	2.52	10.96	<0.001
	FGFR1	HG	6/53 (11)	LG	0/164 (0)	<0.001	Inf	3.90	Inf	0.001
	ERBB2	HG	11/53 (21)	LG	7/164 (4)	0.001	5.81	1.92	18.85	0.003
	CCND1	HG	9/53 (17)	LG	7/164 (4)	0.005	4.55	1.42	15.26	0.016
T1	FGFR3	LG	6/7 (86)	HG	11/46 (24)	0.003	17.87	1.87	897.20	0.02

OR: odds ratio; FDR: false discovery rate; Ref. group: reference group; Comp. group: comparison group. The reference group was the baseline for comparison, while the comparison group was evaluated against it to identify differences.

## Data Availability

The underlying data are not publicly available because of ethical and legal concerns. The anonymized data can be accessed through the Stavanger University Hospital Institutional Data Access/Ethics Committee (contact via email: rek-vest@uib.no, REK vest, Rogaland, Vestland, Norway) by researchers who meet the criteria for accessing confidential data.

## Supplementary Materials

The following supporting information can be downloaded at: https://www.mdpi.com/article/10.3390/diagnostics14192137/s1, Figure S1. (A) and (B) Sections show strong AR positivity (≥50%, P. nr 42) and weak AR positivity (<50%, P. nr 198), respectively. (C) and (D) AR-stained sections were analyzed using the image analysis software Visiopharm®. Green indicates positive cells, while blue indicates negative cells. Scale bar: 200 μm. Figure S2. The Venn diagram shows the distribution of genes with SNVs in red, genes with both SNVs and CNVs in orange, and genes with CNVs alone in yellow. Figure S3. In the mutational signature and enrichment analysis, (A) COSMIC SBS15, 2, 1, 4, 3, 7, 16, and 25 were observed. The y-axis shows the fraction of 96 trinucleotide motifs contributing to the overall signature, while the x-axis indicates the types of SBS across the genome in our cohort. The plot title shows the closest match to validated COSMIC signatures, the cosine similarity value, and the mutational process. (B) In our cohort, we identified eight mutational signatures significantly associated with gene mutations. (C) We used the cophenetic correlation coefficient to determine the optimal number of signatures, which was eight. (D) The mutational signatures were compared to the 30 experimentally validated COSMIC signatures using cosine similarity values. Table S1. Samples without AR genetic alterations exhibited IHC AR negativity, while those with AR genetic alterations demonstrated AR positivity. The threshold for IHC positivity was set at 50%. Table S2. Summary of 411 SNVs and INDELs. Table S3. Summary of 193 CNVs.
