# Supplementary material for: Integrating Genetic Alterations and Histopathological Features for Enhanced Risk Stratification in Non-Muscle-Invasive Bladder Cancer
Source: Diagnostics (Basel). 2024 Sep 26;14(19):2137. doi: 10.3390/diagnostics14192137 (PMC11482629; doi:10.3390/diagnostics14192137)
Supplement: Supplementary file 1 [file diagnostics-14-02137-s001.zip › Revised Supplementary material.pdf]

Supplementary information

Integrating genetic alterations and histopathological features for enhanced risk stratification in non-muscle invasive bladder cancer

Melinda Lillesand[1,2*], Vebjørn Kvikstad[3], Einar Gudlaugsson[1], Ivar Skaland[1], Aida Slewa Johannessen[1], Almaz Nigatu Tesfahun[1], Sigmund Vegard Sperstad[1], Emiel A.M. Janssen[1,2#], Marie Austdal[1,4#]

1 Department of Pathology, Stavanger University Hospital, Stavanger, Norway
2 Department of Chemistry, Bioscience and Environmental Engineering, University of Stavanger, Stavanger, Norway
3 Department of Forensic Medicine, Oslo University Hospital, Oslo, Norway
4 Department of Research, Section for Biostatistics, Stavanger University Hospital, Stavanger, Norway
* Correspondence: melinda.lillesand@sus.no
# These authors share last authorship

Figure S1 2

Table S1 2

Figure S2 3

Figure S3 4-6

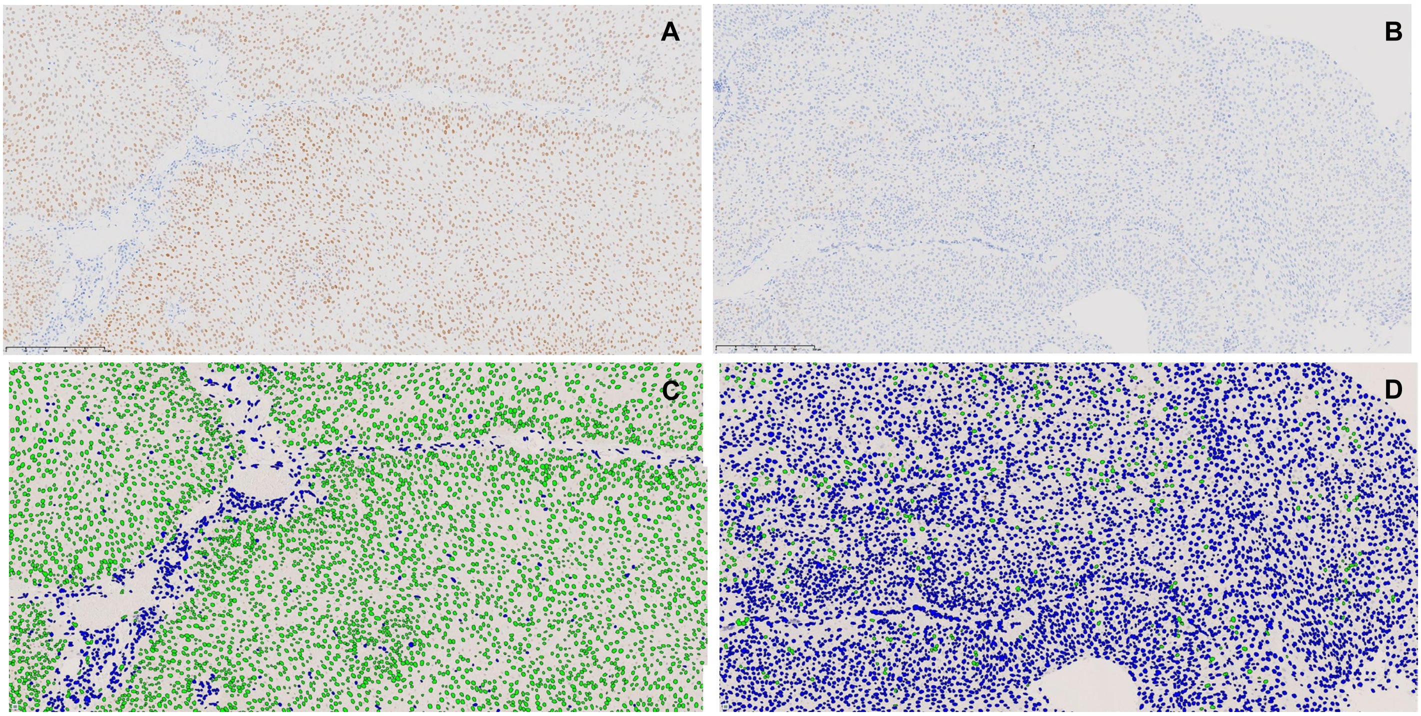

Figure S1. (A) and (B) Sections show strong AR positivity (≥50%, P. nr 42) and weak AR positivity (<50%, P. nr 198), respectively. (C) and (D) AR-stained sections were analyzed using the image analysis software Visiopharm®. Green indicates positive cells, while blue indicates negative cells. Scale bar: 200 μm.

Table S1. Samples without AR genetic alterations exhibited IHC AR negativity, while those with AR genetic alterations demonstrated AR positivity. The threshold for IHC positivity was set at 50%.

P. nr.	NGS AR+	IHC AR%	Stage	WHO04 grade	Recurrence	Progression	Sex
198	No	4.1	T1	Low	No	No	Female
202	No	28.6	Ta	High	No	No	Male
207	No	8.1	T1	High	No	No	Male
227	No	6.1	T1	High	No	No	Male
240	No	5.1	T1	High	No	No	Male
242	No	3.8	Ta	High	No	No	Female
268	No	2.7	Ta	High	No	No	Male
23	Yes	91.9	Ta	Low	Yes	No	Female
42	Yes	79.1	Ta	High	Yes	No	Female
158	Yes	66.4	T1	High	Yes	No	Female
199	Yes	57.9	Ta	High	No	No	Female
225	Yes	84.3	Ta	Low	No	No	Female
226	Yes	57.3	T1	High	No	No	Female
349	Yes	0.4	T1	High	Yes	No	Female

* P. nr.: Project number

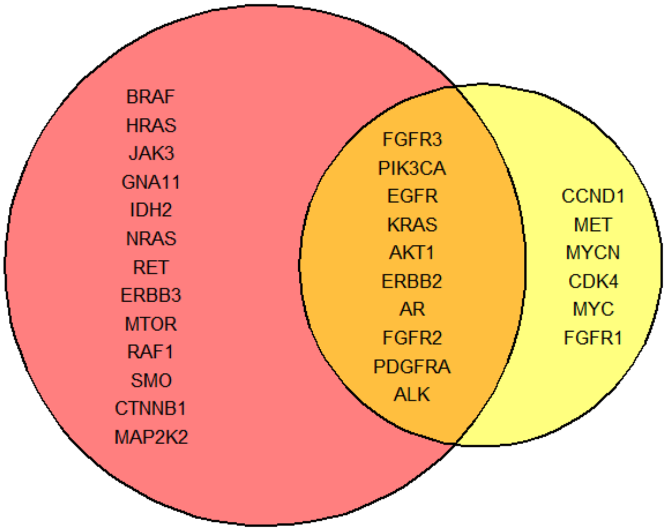

Figure S2. The Venn diagram shows the distribution of genes with SNVs in red, genes with both SNVs and CNVs in orange, and genes with CNVs alone in yellow.

A.

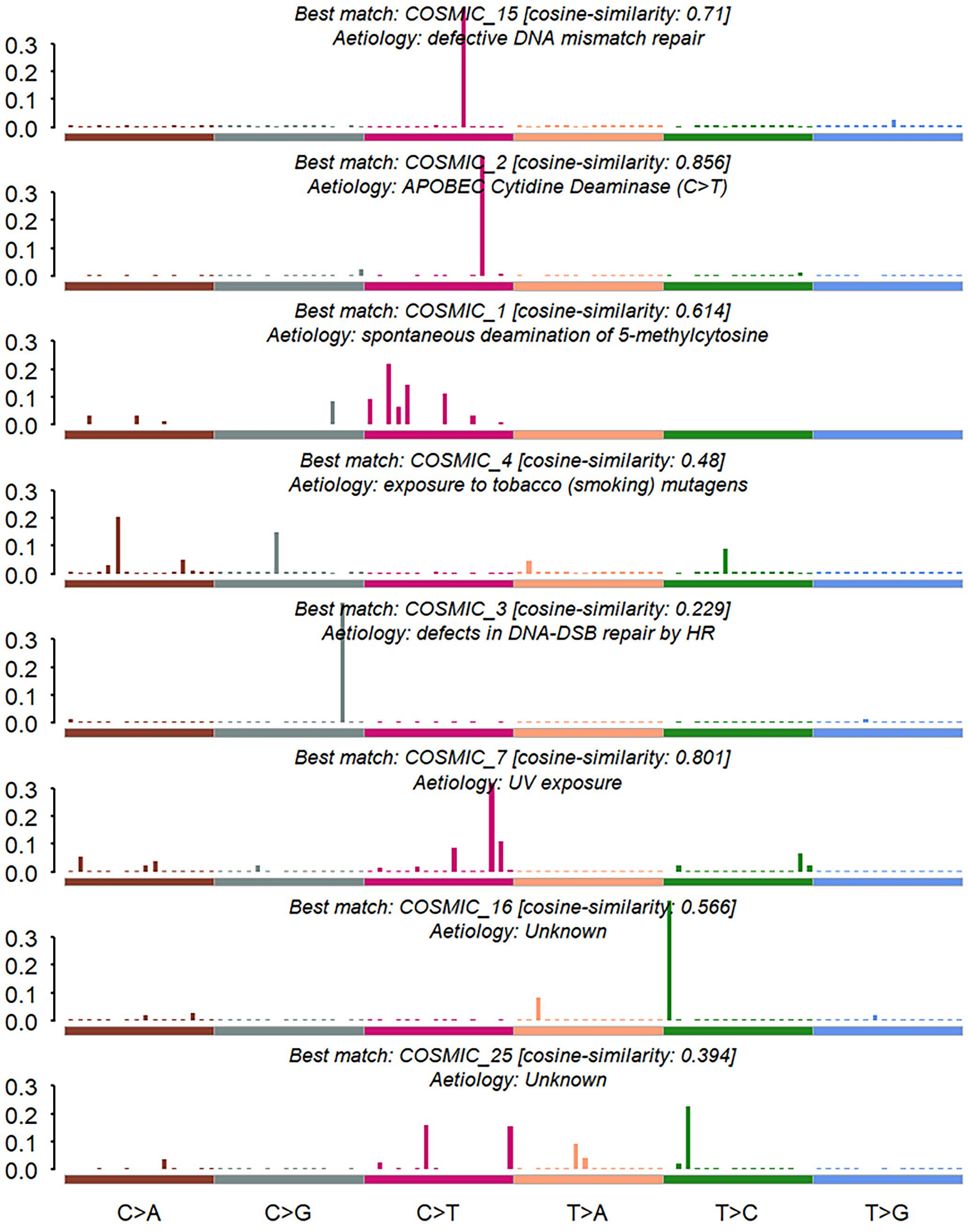
Best match: COSMIC_15 [cosine-similarity: 0.71]
Aetiology: defective DNA mismatch repair
Best match: COSMIC_2 [cosine-similarity: 0.856]
Aetiology: APOBEC Cytidine Deaminase (C>T)
Best match: COSMIC_1 [cosine-similarity: 0.614]
Aetiology: spontaneous deamination of 5-methylcytosine
Best match: COSMIC_4 [cosine-similarity: 0.48]
Aetiology: exposure to tobacco (smoking) mutagens
Best match: COSMIC_3 [cosine-similarity: 0.229]
Aetiology: defects in DNA-DSB repair by HR
Best match: COSMIC_7 [cosine-similarity: 0.801]
Aetiology: UV exposure
Best match: COSMIC_16 [cosine-similarity: 0.566]
Aetiology: Unknown
Best match: COSMIC_25 [cosine-similarity: 0.394]
Aetiology: Unknown
0.3
0.2
0.1
0.0
C>A
C>G
C>T
T>A
T>C
T>G

B.

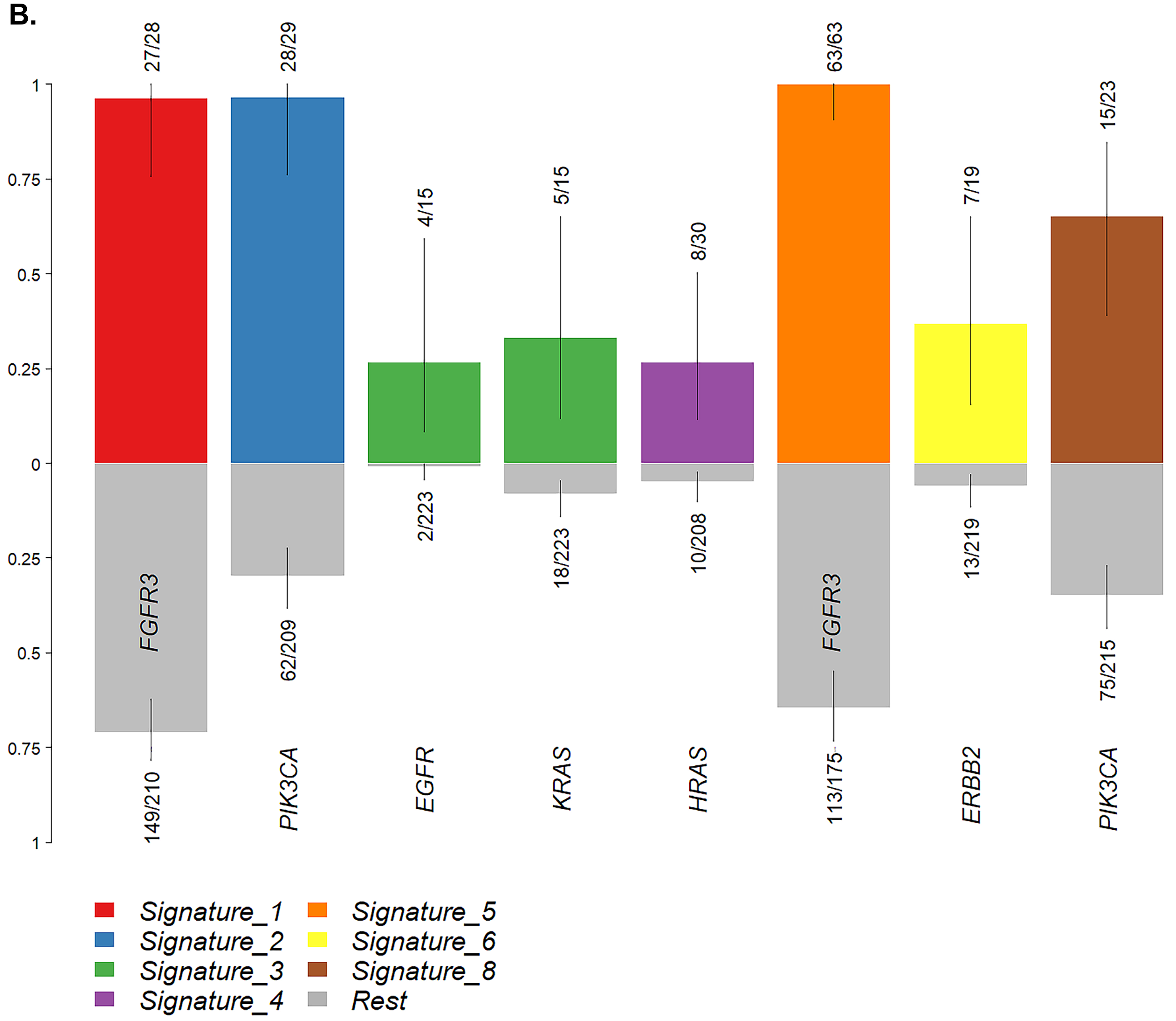
27/28
28/29
4/15
5/15
8/30
63/63
7/19
15/23
1
0.75
0.5
0.25
0
0.25
0.5
0.75
1
2/223
18/223
10/208
13/219
FGFR3
62/209
FGFR3
75/215
149/210
PIK3CA
EGFR
KRAS
HRAS
113/175
ERBB2
PIK3CA
Signature_1
Signature_2
Signature_3
Signature_4
Signature_5
Signature_6
Signature_8
Rest

C.

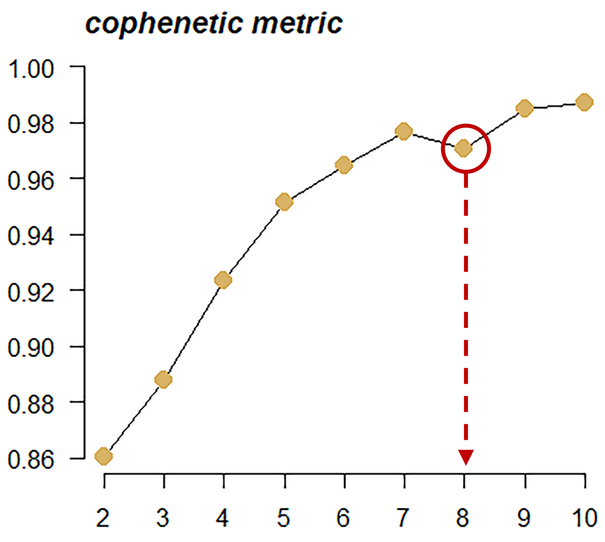
cophenetic metric
1.00
0.98
0.96
0.94
0.92
0.90
0.88
0.86
2
3
4
5
6
7
8
9
10

D.

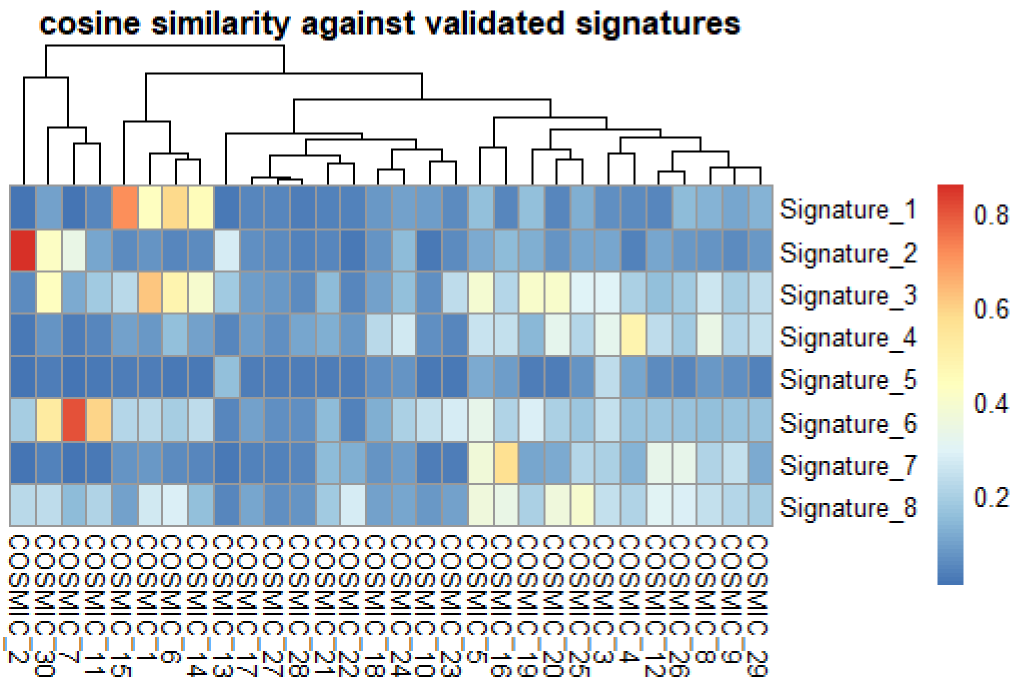

Figure S3. In the mutational signature and enrichment analysis, (A) COSMIC SBS15, 2, 1, 4, 3, 7, 16, and 25 were observed. The y-axis shows the fraction of 96 trinucleotide motifs contributing to the overall signature, while the x-axis indicates the types of SBS across the genome in our cohort. The plot title shows the closest match to validated COSMIC signatures, the cosine similarity value, and the mutational process. (B) In our cohort, we identified eight mutational signatures significantly associated with gene mutations. (C) We used the cophenetic correlation coefficient to determine the optimal number of signatures, which was eight. (D) The mutational signatures were compared to the 30 experimentally validated COSMIC signatures using cosine similarity values.
